# Supplementary material for: A Streamlined High-Throughput LC-MS Assay for Quantifying Peptide Degradation in Cell Culture
Source: bioRxiv. 2024 Oct 15:2024.10.11.617883. Preprint. [Version 1] doi: 10.1101/2024.10.11.617883 (PMC11507709; doi:10.1101/2024.10.11.617883)
Supplement: Supplement 1 [file media-1.pdf]

A Streamlined High-Throughput LC-MS Assay for Quantifying Peptide Degradation in Cell Culture

Samuel J. Rozans[a], Abolfazl S. Moghaddam, E. Thomas Pashuck[a]

[a]Department of Bioengineering, Lehigh University, Bethlehem, PA, USA, 18015.

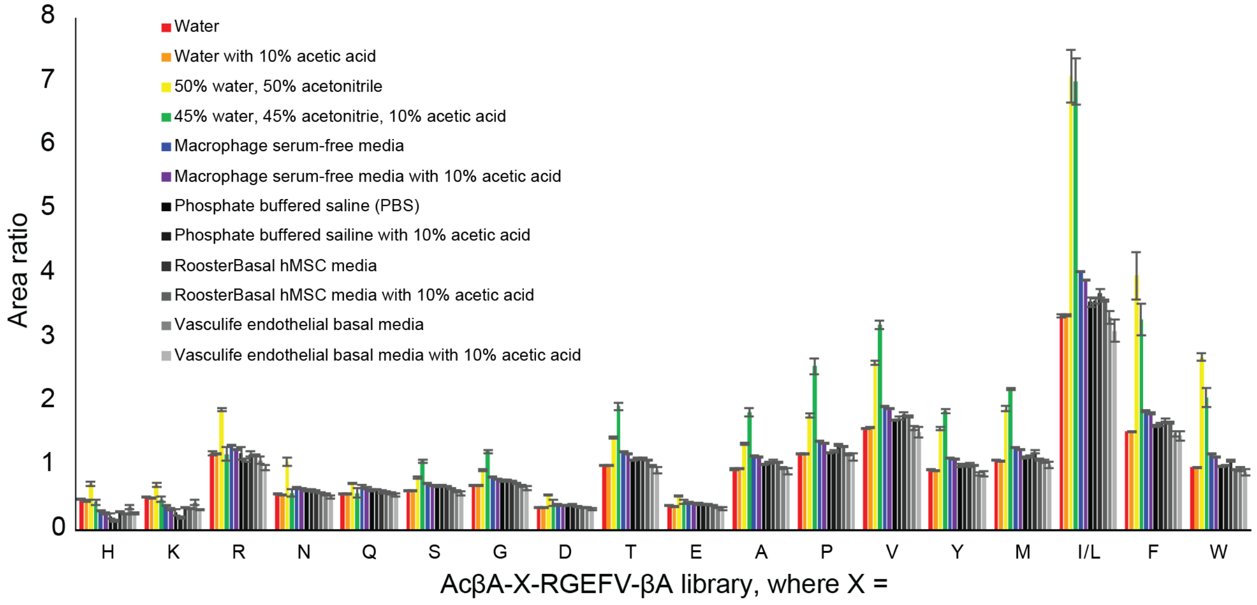

Figure S1. Degradation of the AcβA-X-RGEFV-βA peptide library in a variety of solvent conditions.

A **Ac-βA-Ala-RGEFV-NH$_2$**

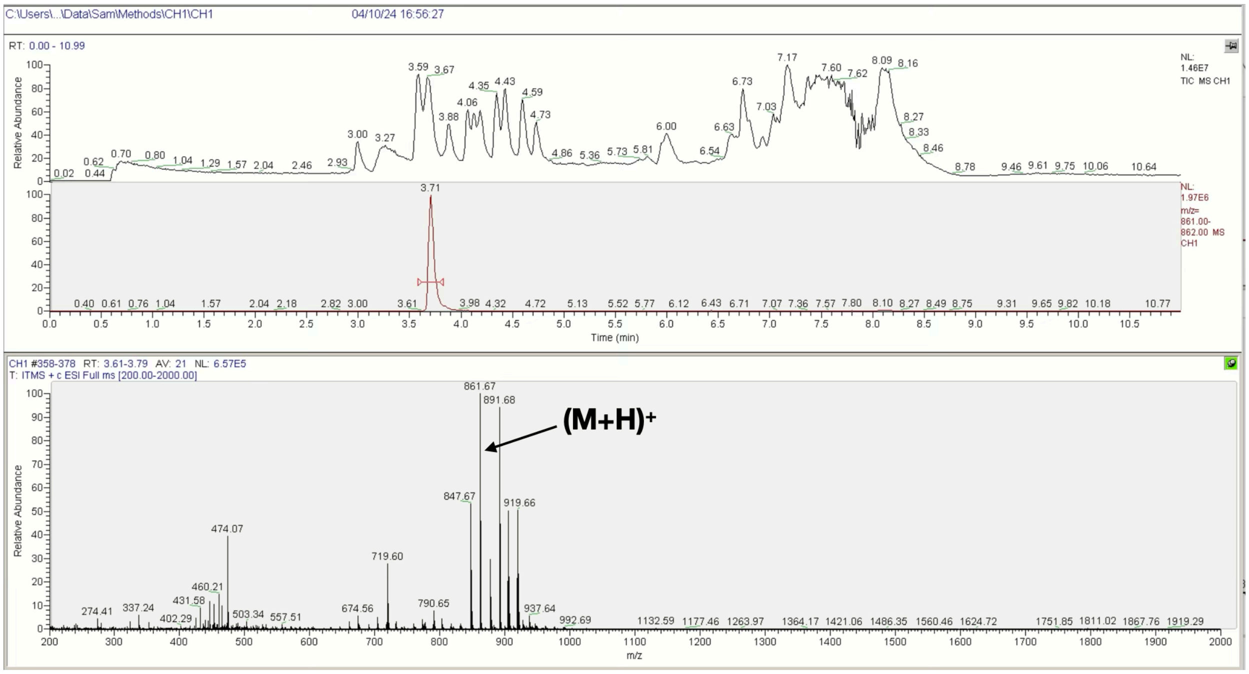

B **Ac-βA-Arg-RGEFV-βA-NH$_2$**

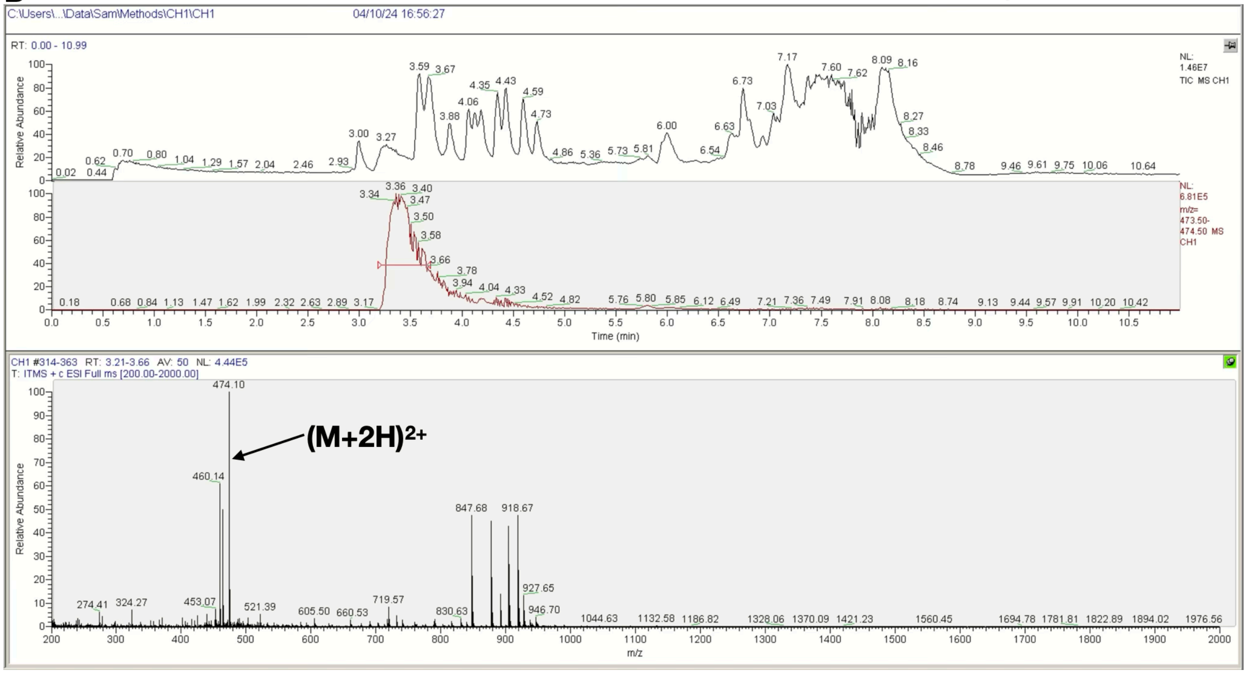

C **Ac-βA-Asn-RGEFV-βA-NH2**

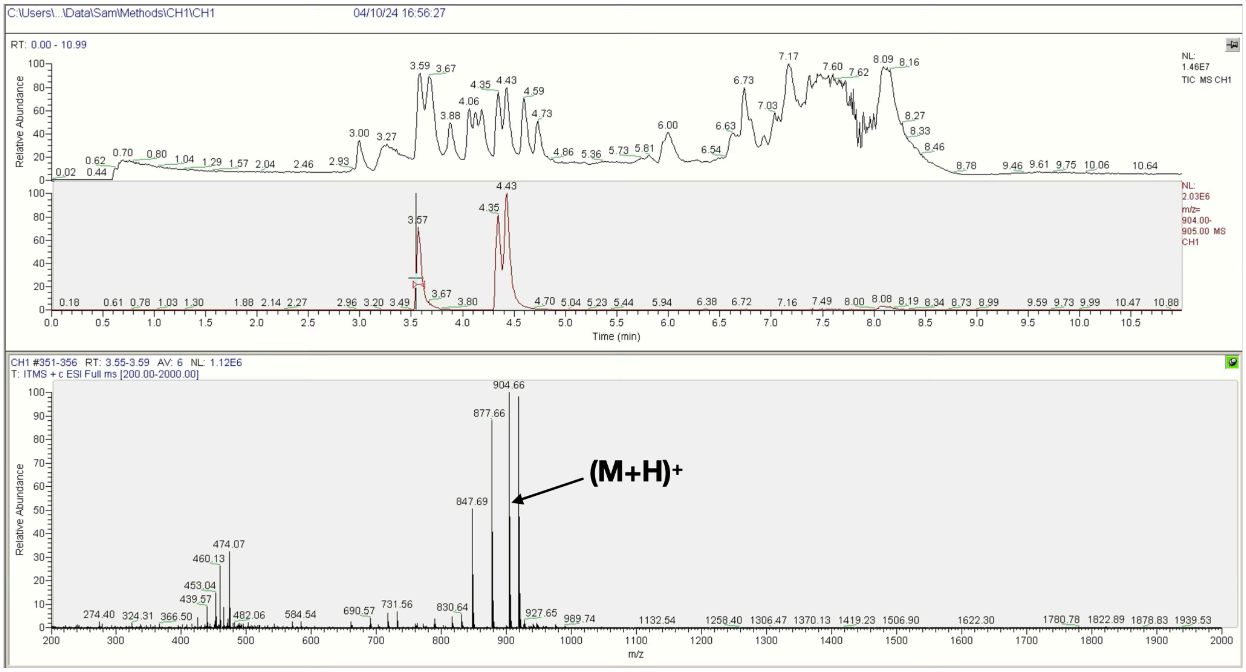

D **Ac-βA-Asp-RGEFV-βA-NH2**

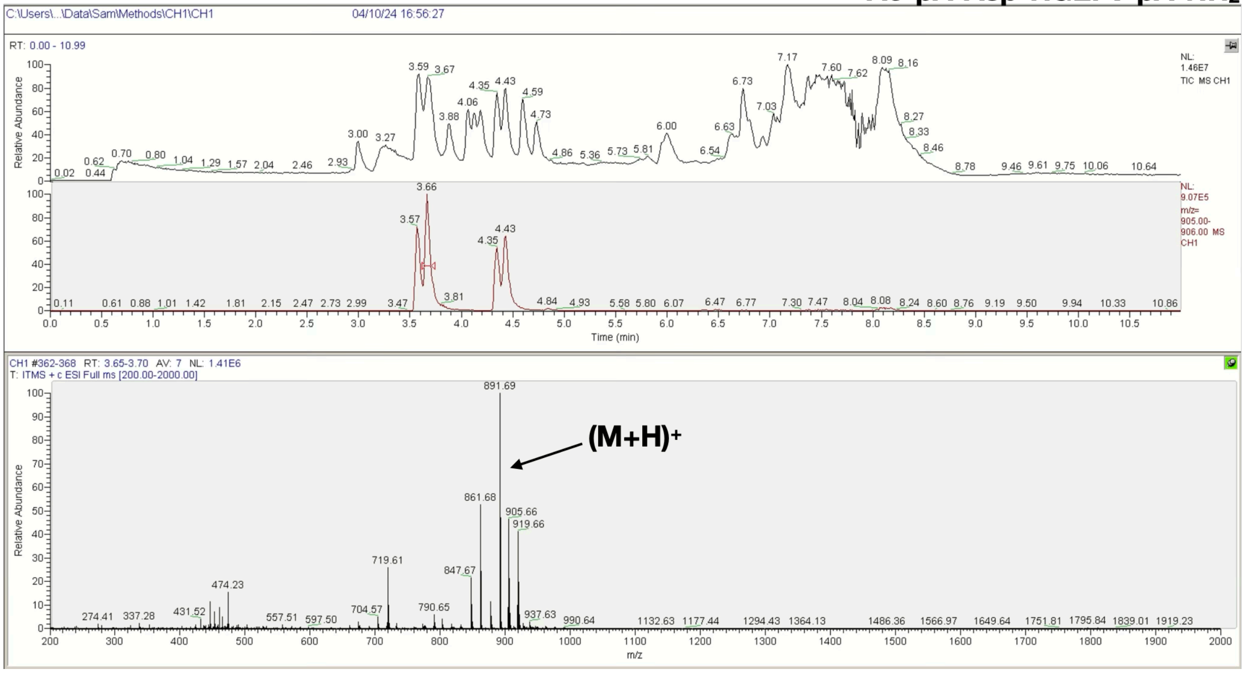

E **Ac-βA-Gln-RGEFV-βA-NH$_2$**

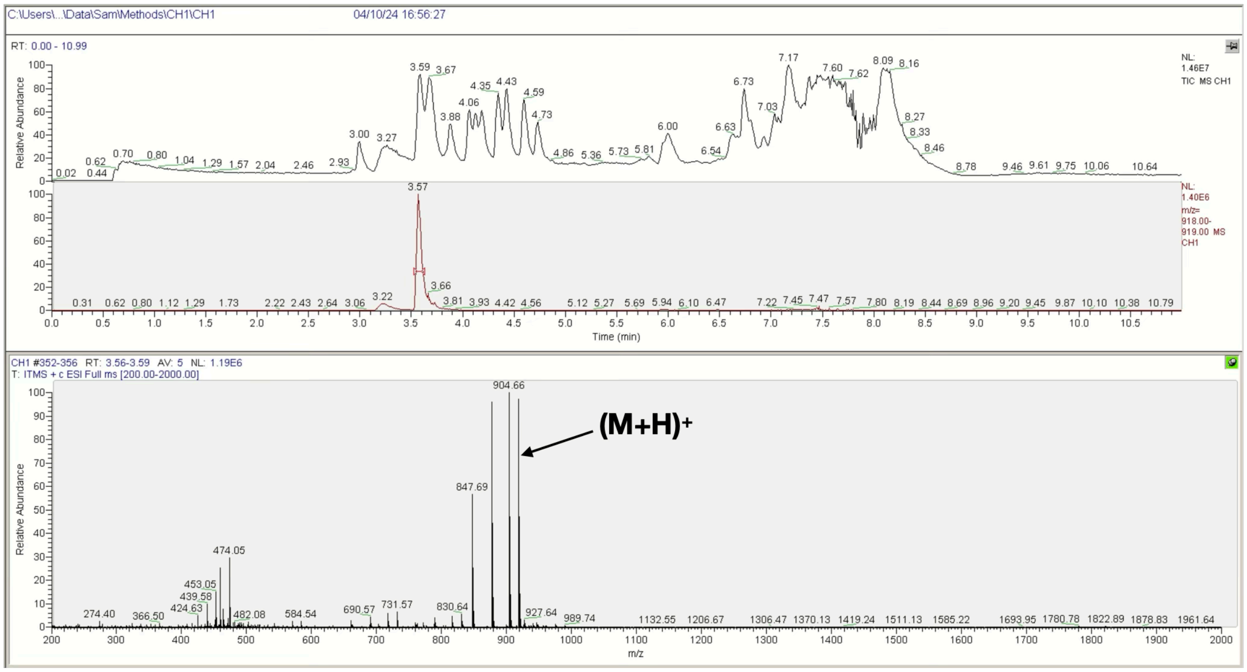

F **Ac-βA-Glu-RGEFV-βA-NH$_2$**

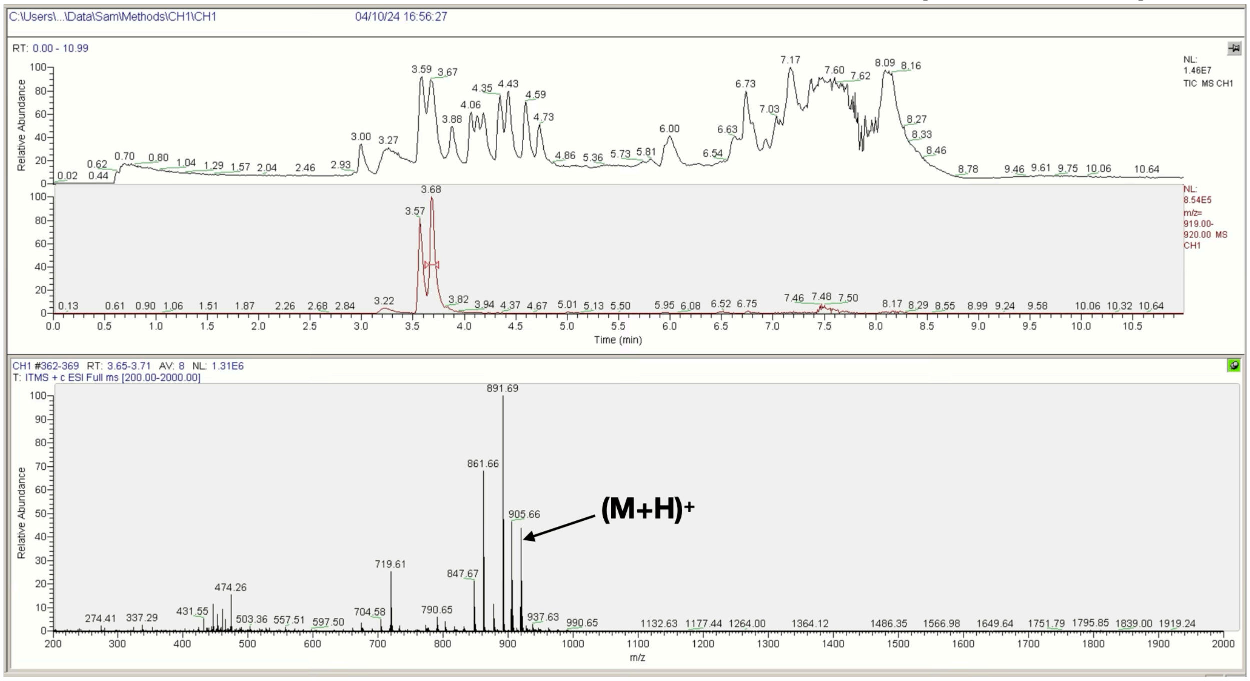

G **Ac-βA-Gly-RGEFV-βA-NH$_2$**

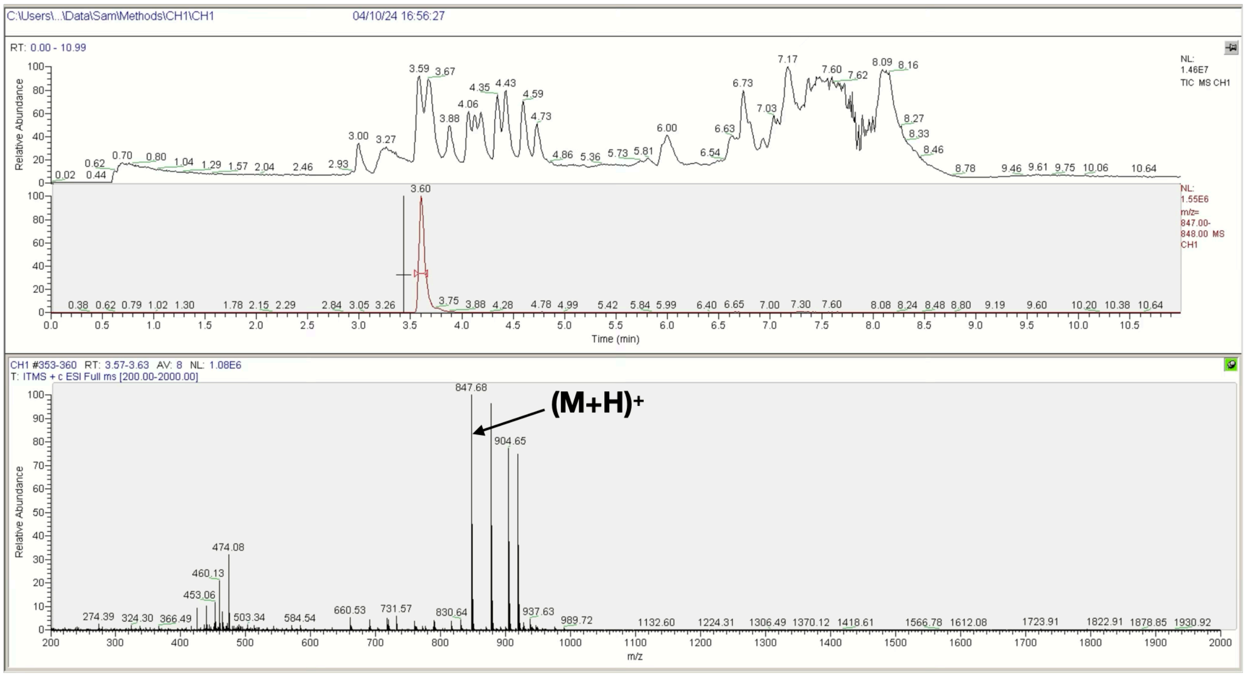

H **Ac-βA-His-RGEFV-βA-NH$_2$**

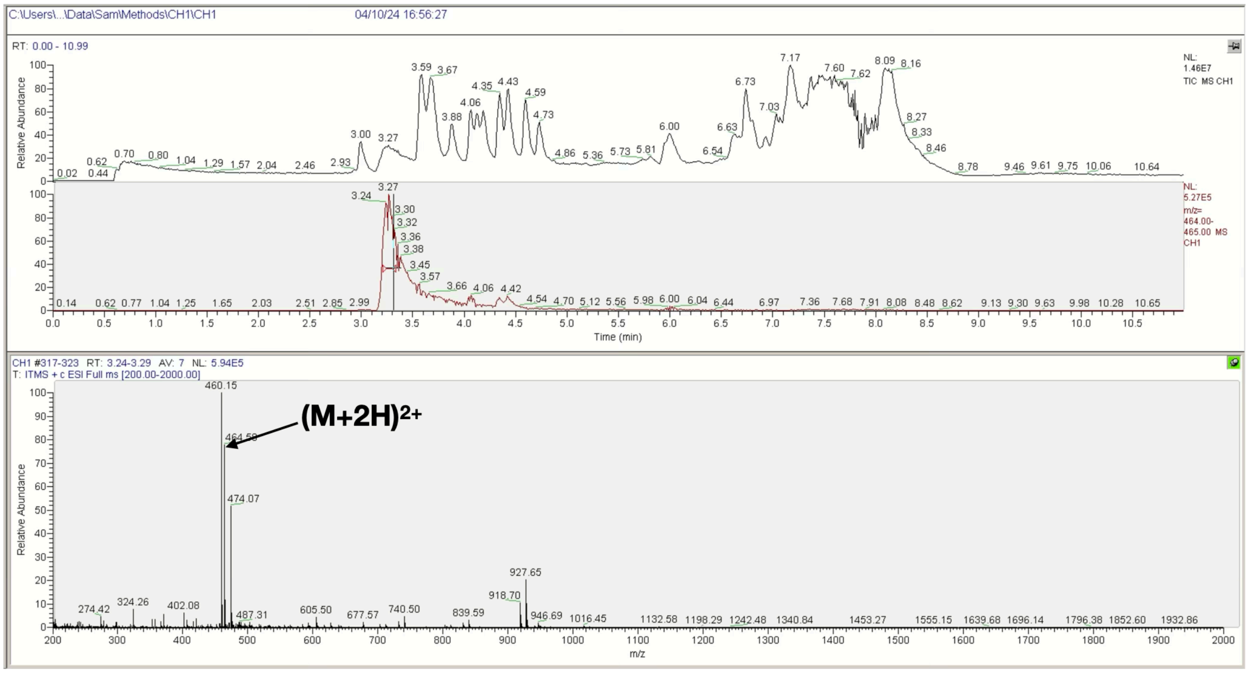

I **Ac-βA-Ile/Leu-RGEFV-βA-NH_2**

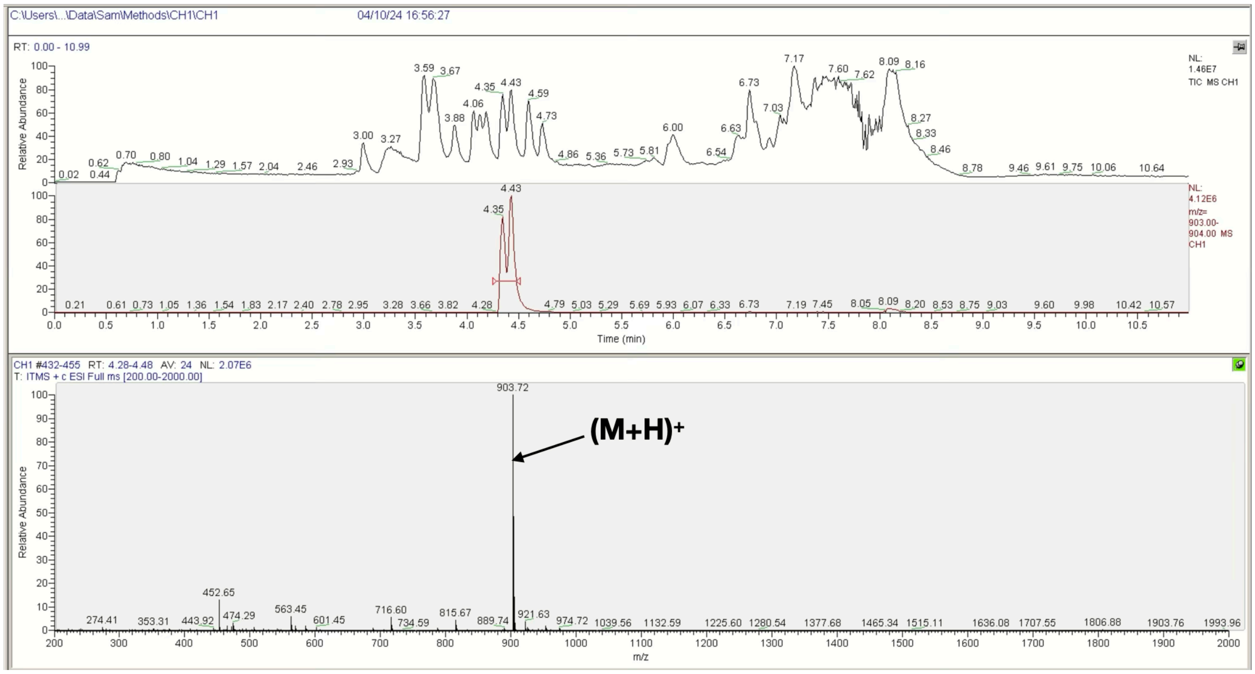

J **Ac-βA-Lys-RGEFV-βA-NH_2**

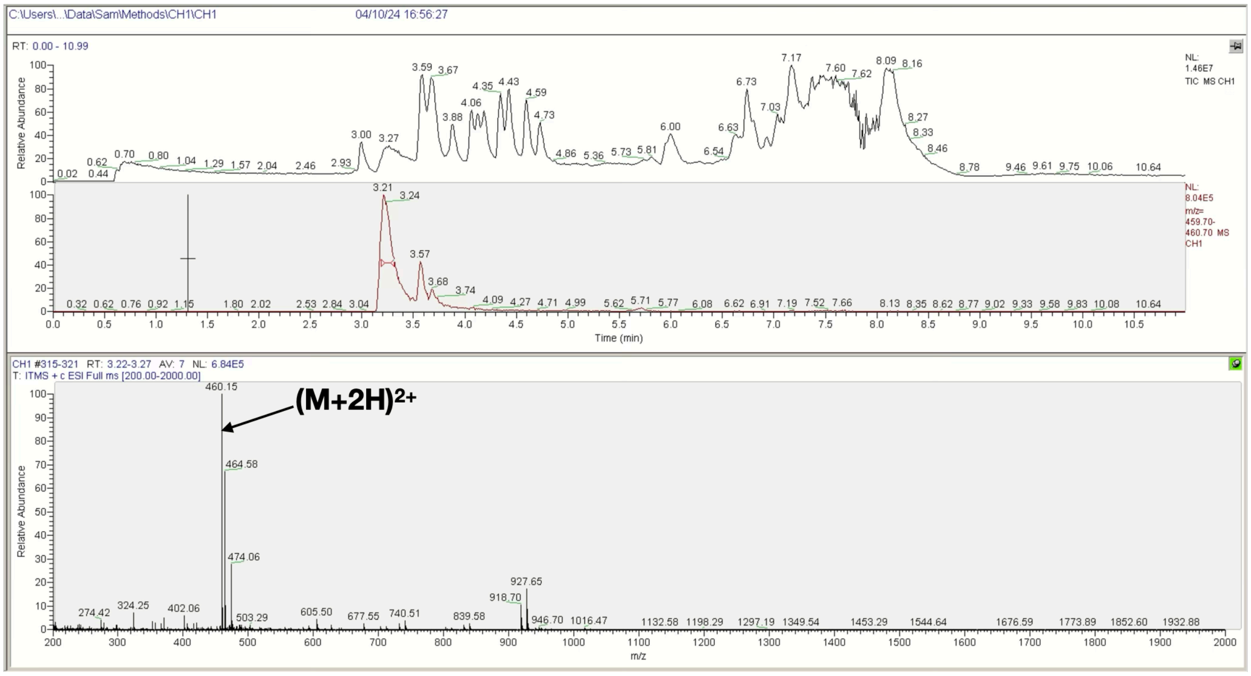

K **Ac-βA-Met-RGEFV-βA-NH₂**

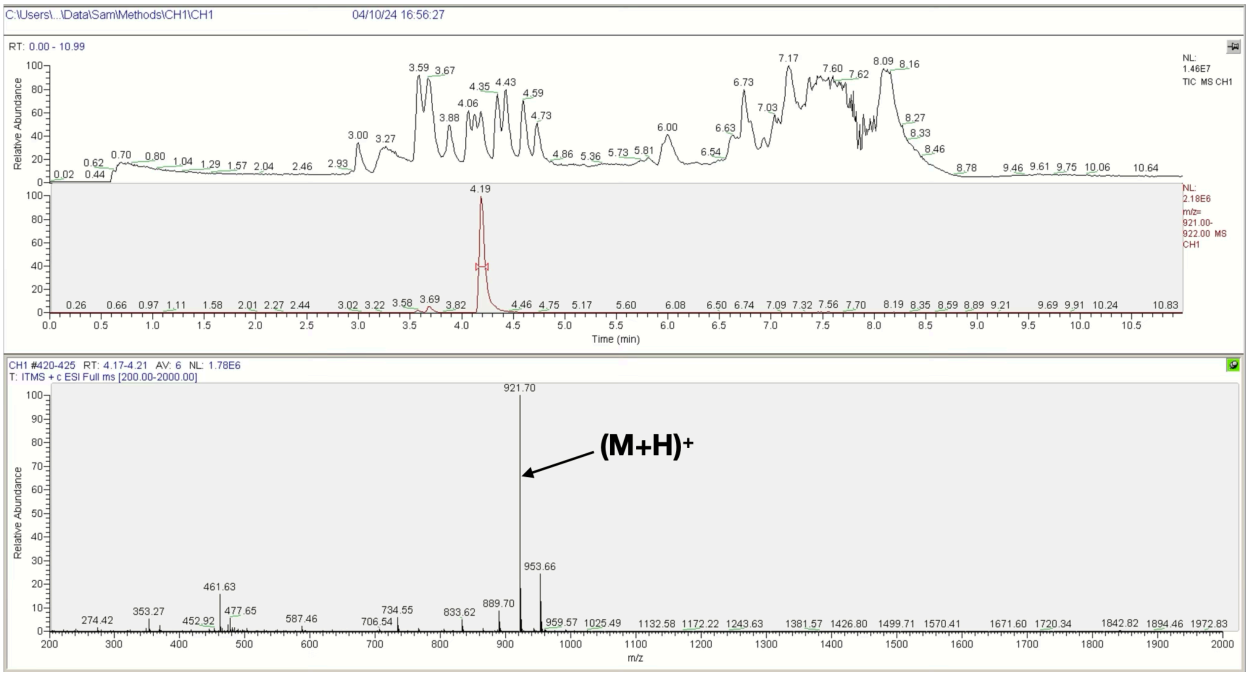

L **Ac-βA-Phe-RGEFV-βA-NH₂**

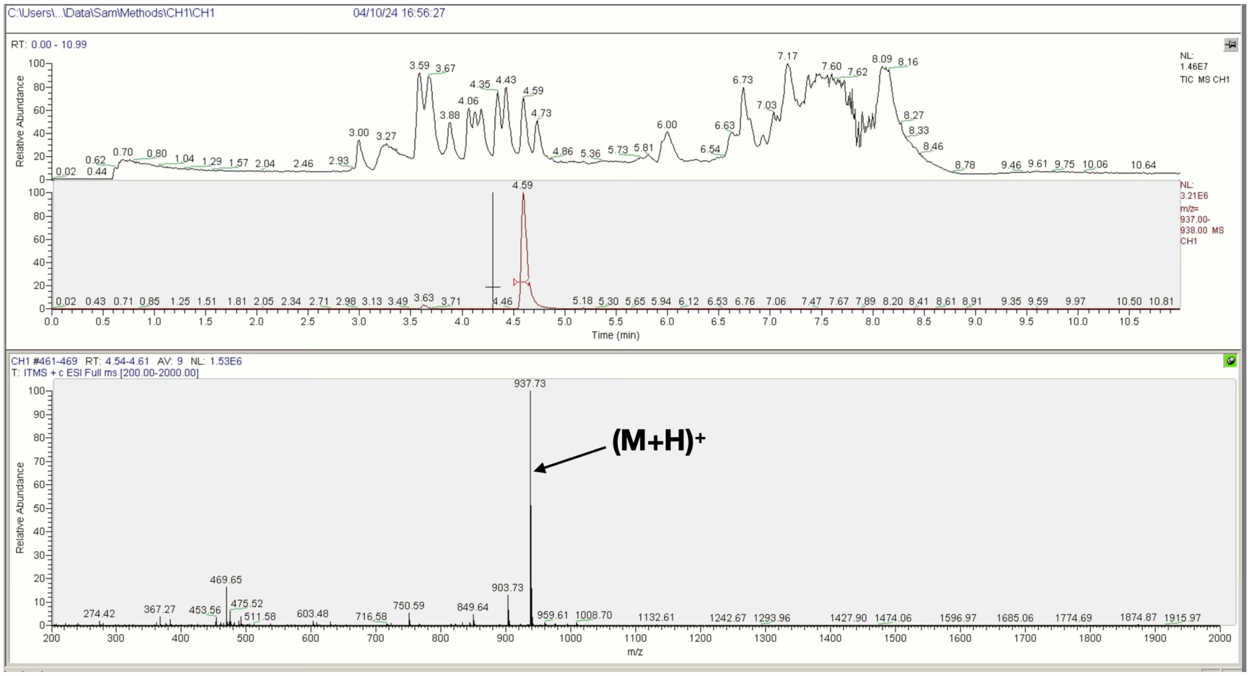

M **Ac-βA-Pro-RGEFV-βA-NH_2**

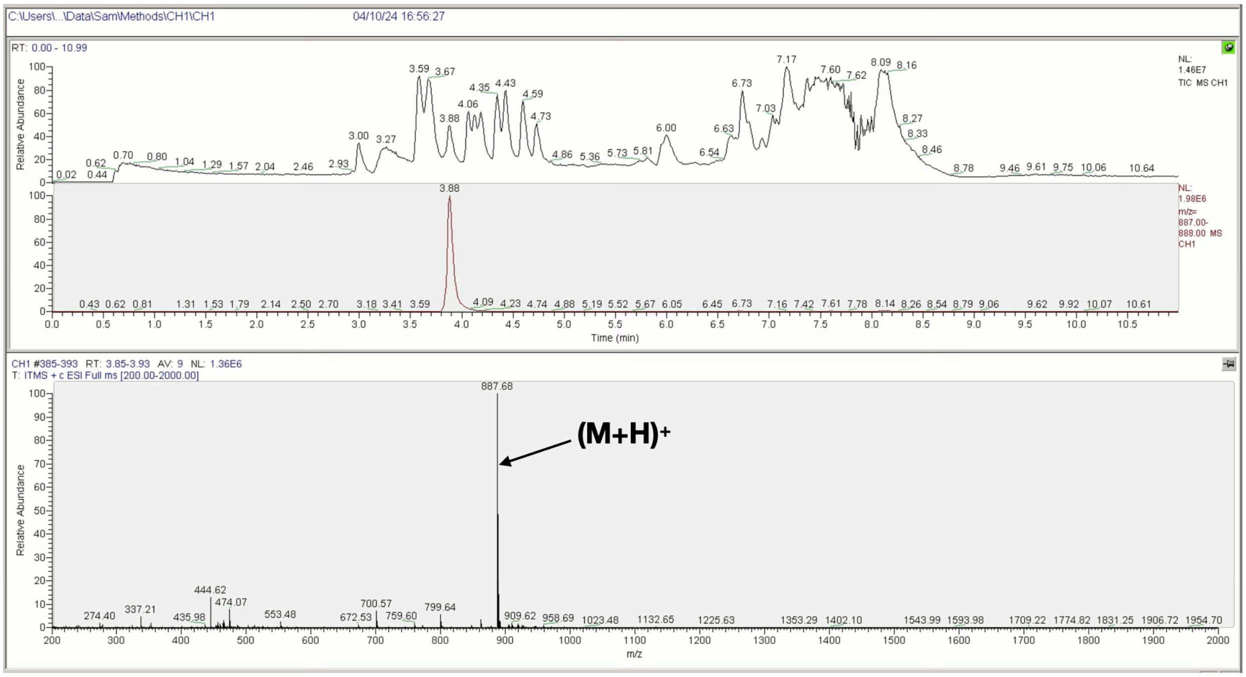

N **Ac-βA-Ser-RGEFV-βA-NH_2**

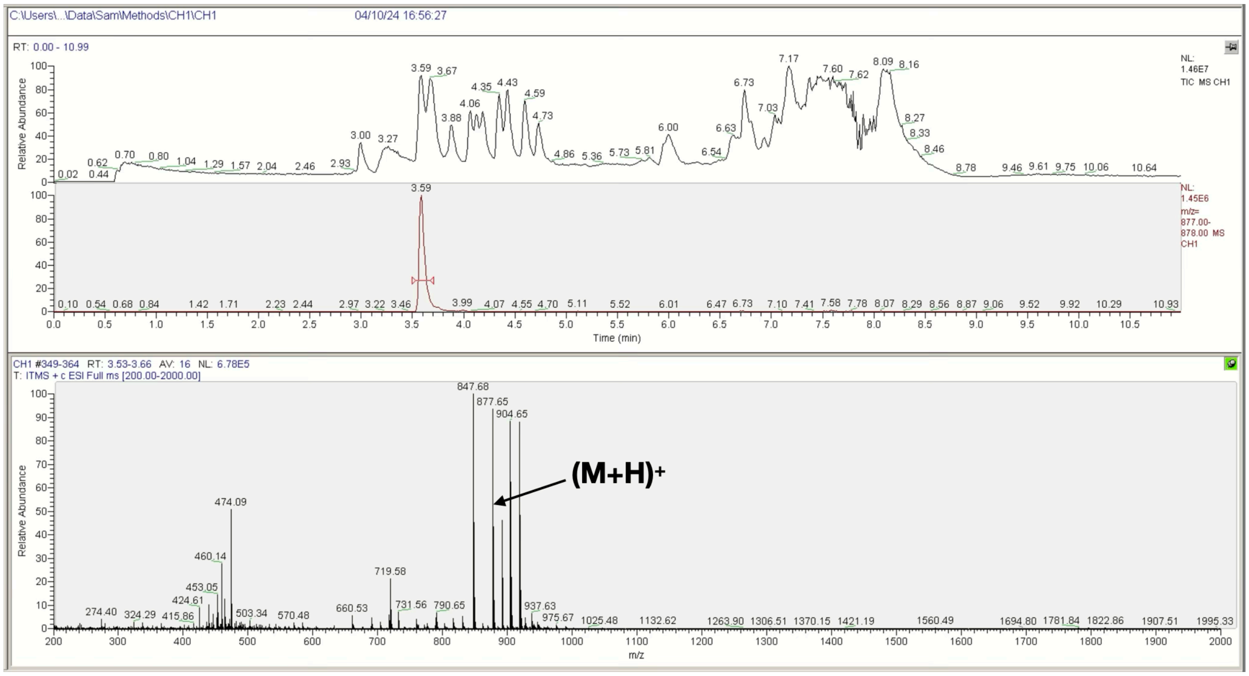

O **Ac-βA-Thr-RGEFV-βA-NH2**

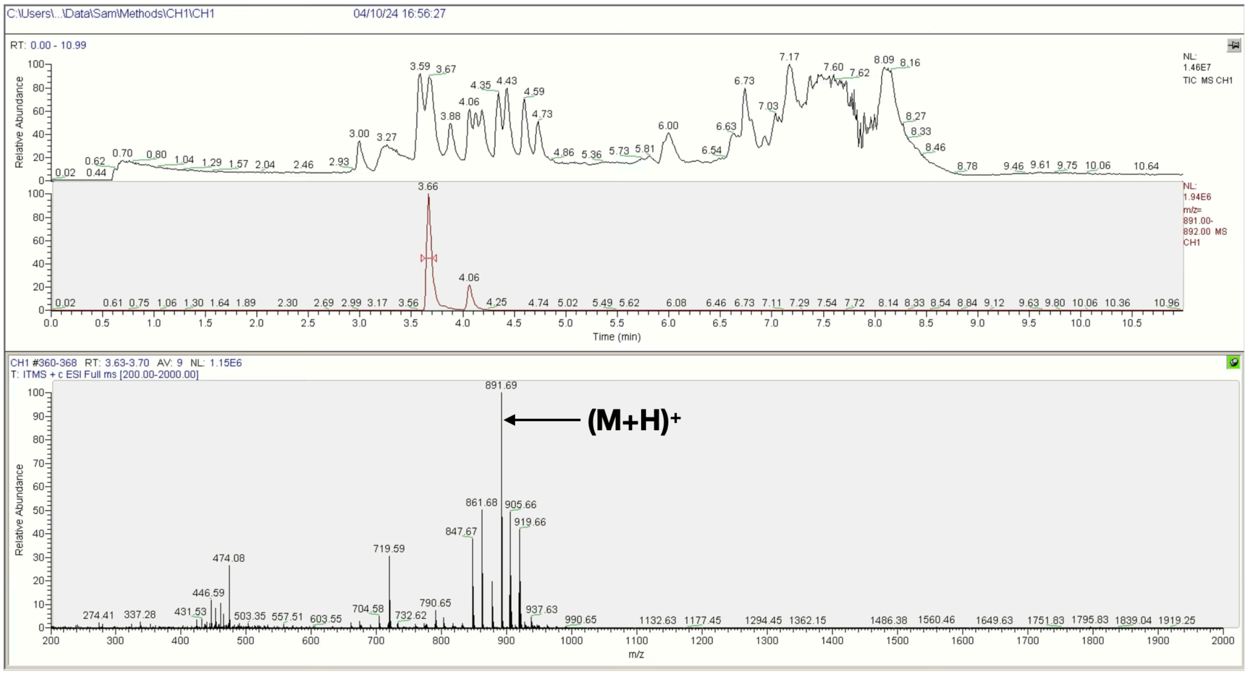

P **Ac-βA-Trp-RGEFV-βA-NH2**

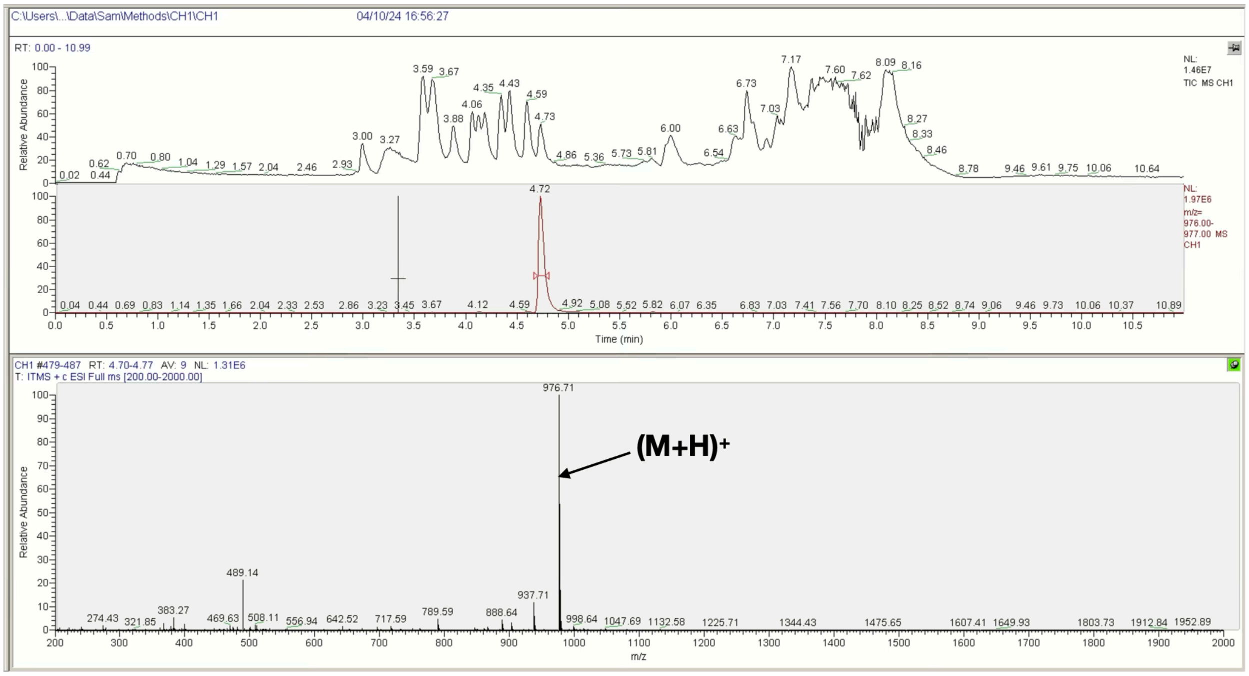

Q **Ac-βA-Tyr-RGEFV-βA-NH$_2$**

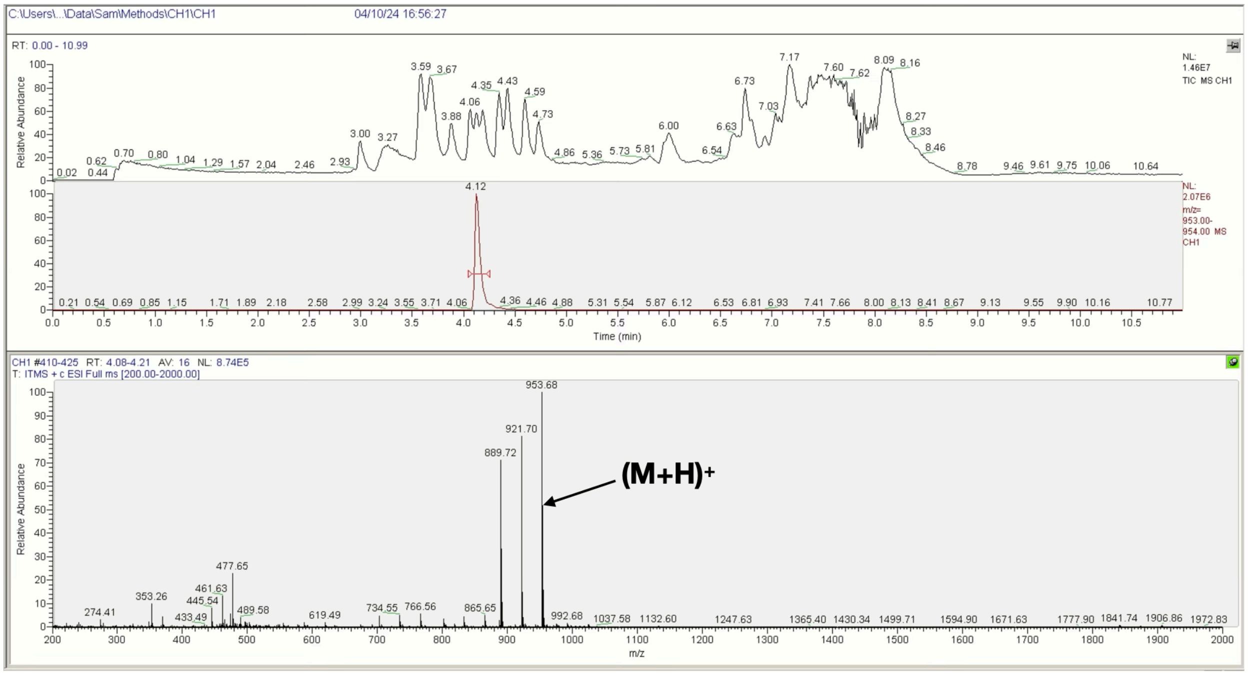

R **Ac-βA-Val-RGEFV-βA-NH$_2$**

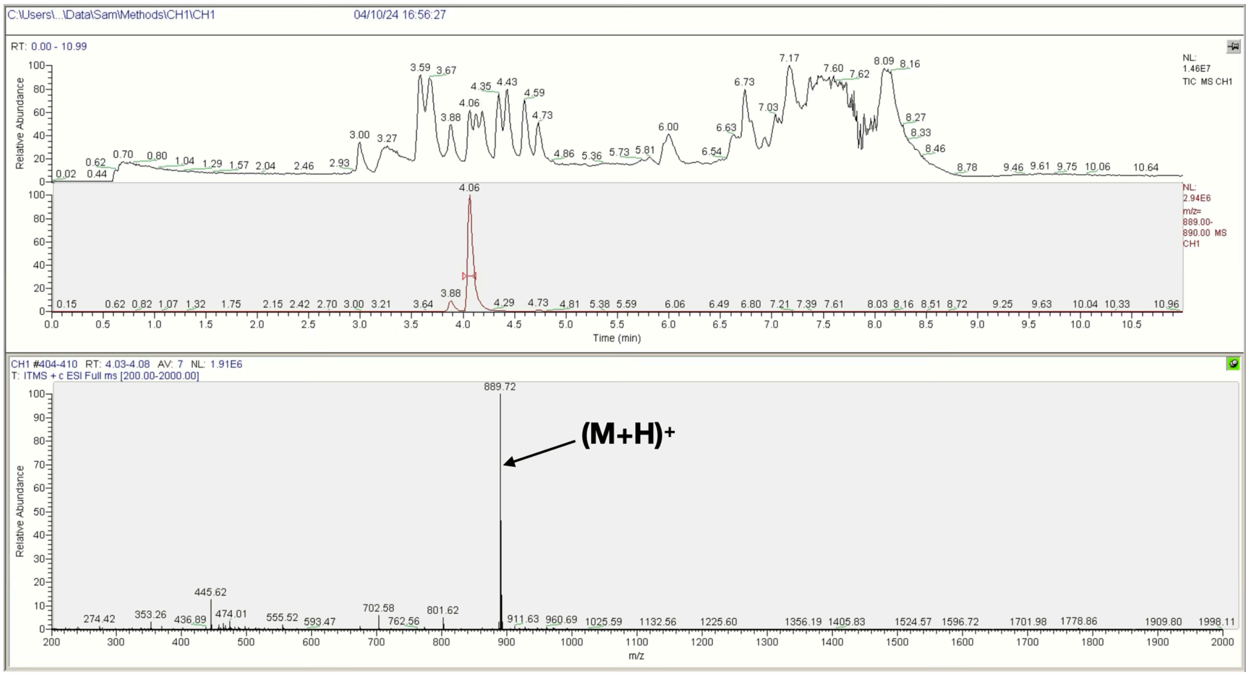

Figure S2. LCMS spectra of the Ac-βA-X-RGEFV-NH$_2$ libraries, where X = a) Ala, b) Arg, c) Asn, d) Asp, e) Gln, f) Glu, g) Gly, h) His, i) Ile/Leu, j) Lys, k) Met, l) Phe, m) Pro, n) Ser, o) Thr, p) Trp, q) Tyr, r) Val.

A **Ac-βA-RGEFV-Ala-βA-NH₂**

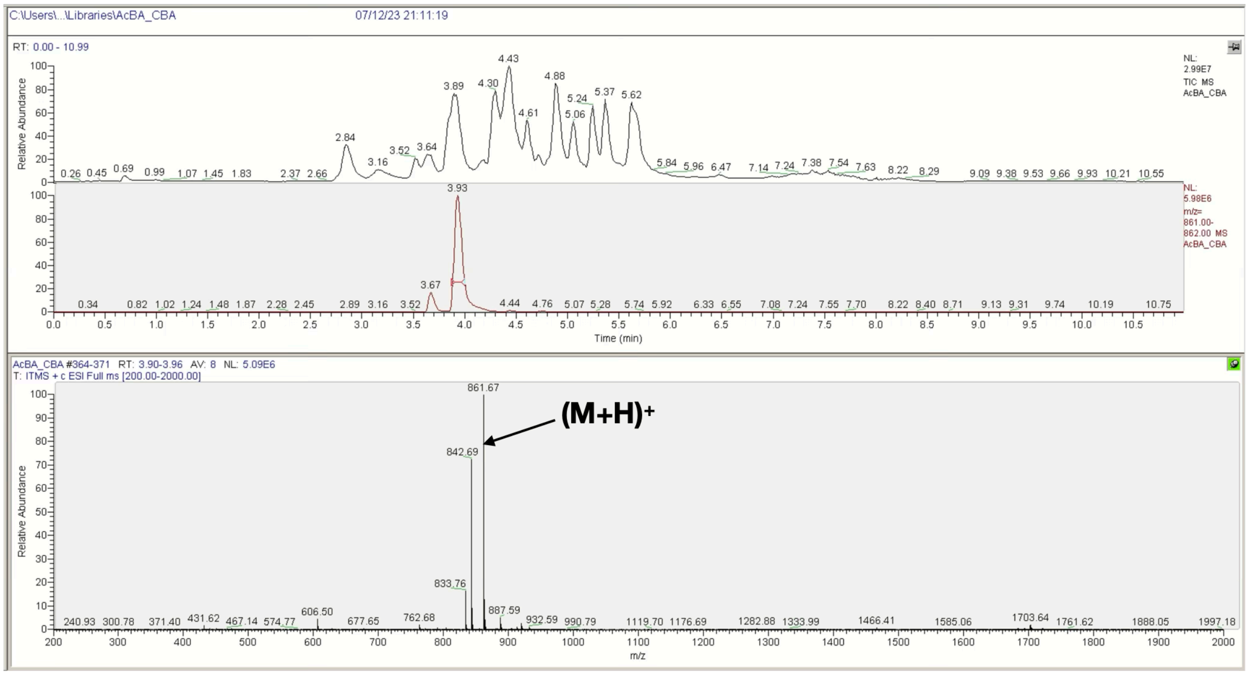

B **Ac-βA-RGEFV-Arg-βA-NH₂**

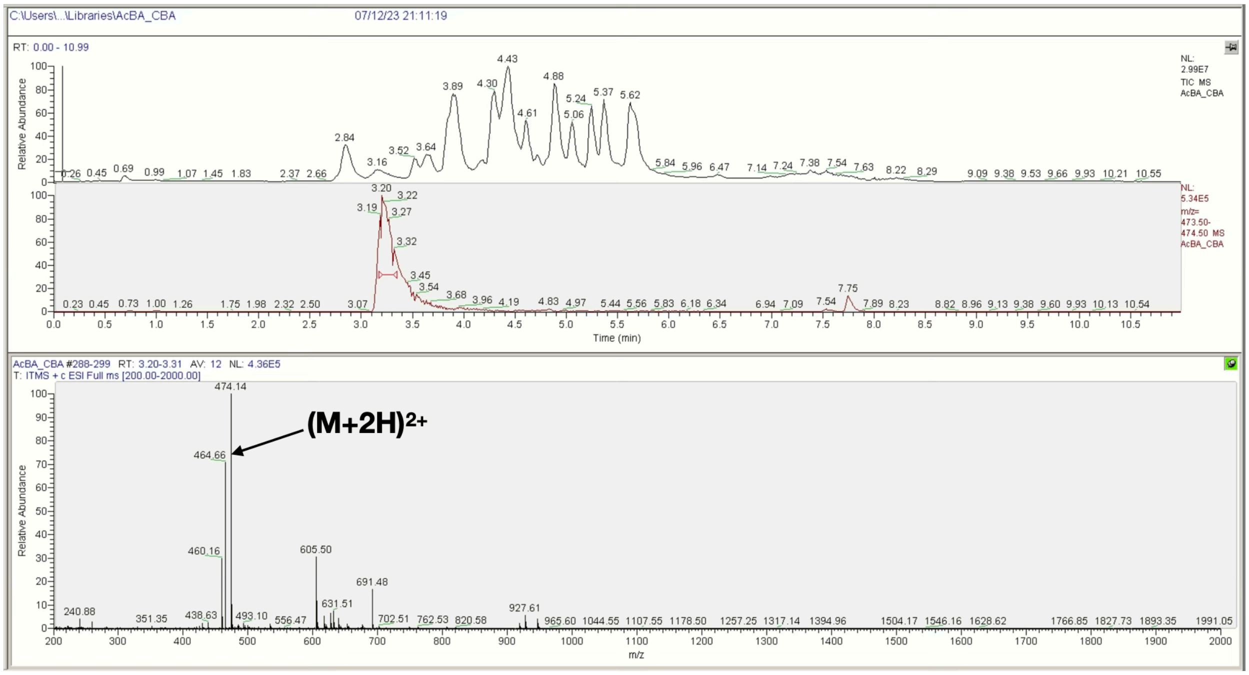

C **Ac-βA-RGEFV-Asn-βA-NH₂**

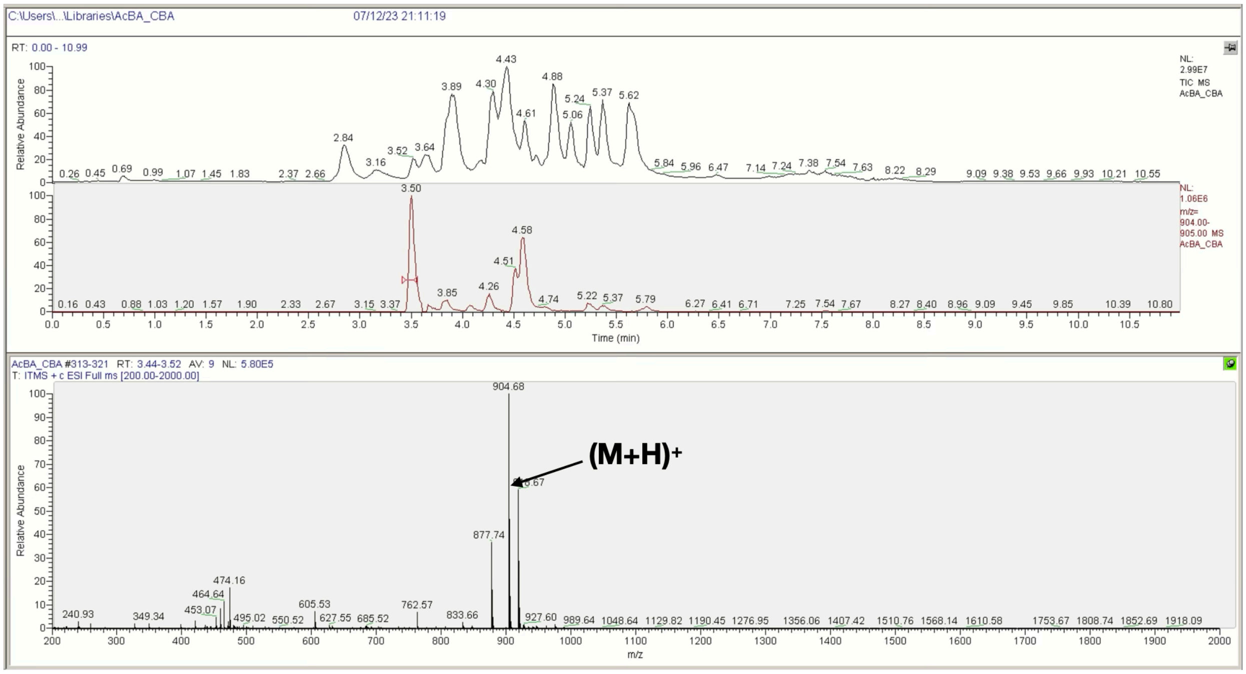

D **Ac-βA-RGEFV-Asp-βA-NH₂**

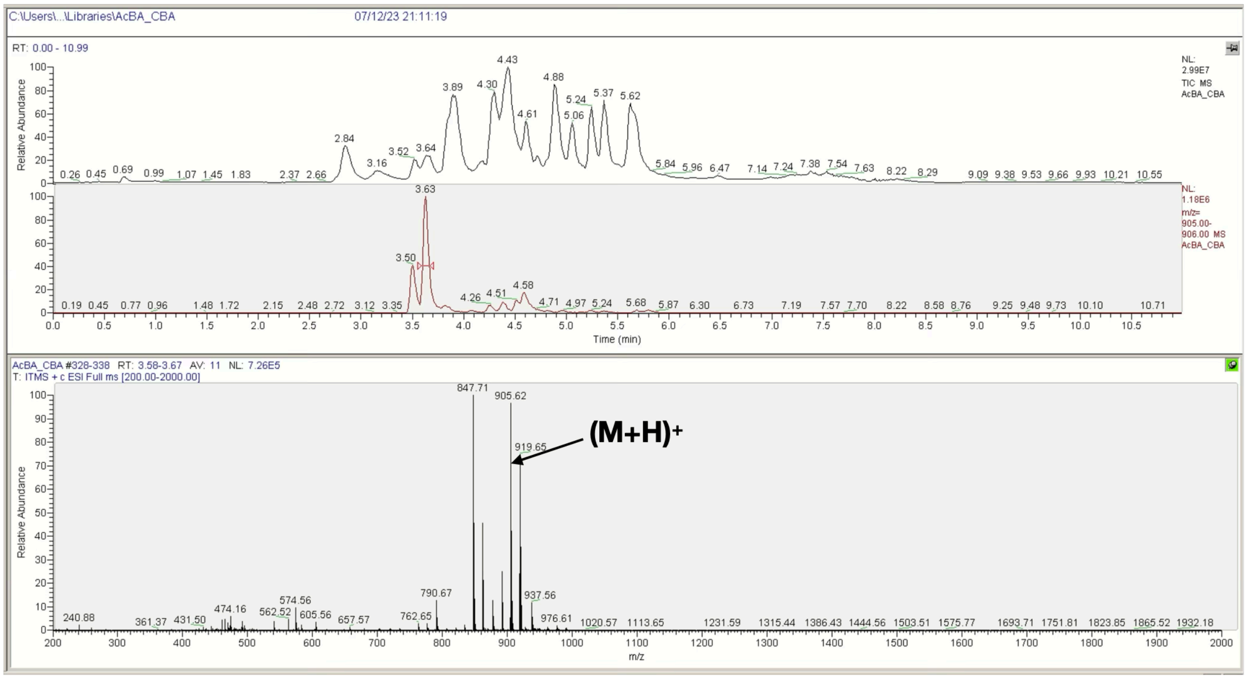

E **Ac-βA-RGEFV-Gln-βA-NH$_2$**

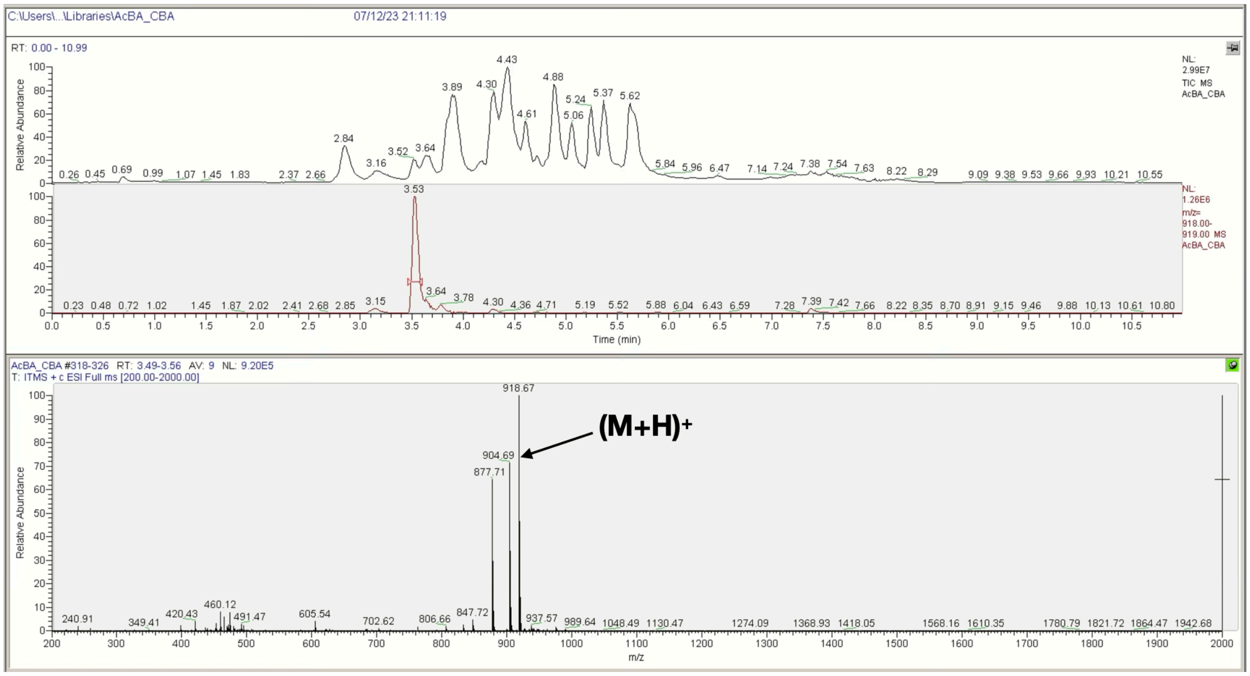

F **Ac-βA-RGEFV-Glu-βA-NH$_2$**

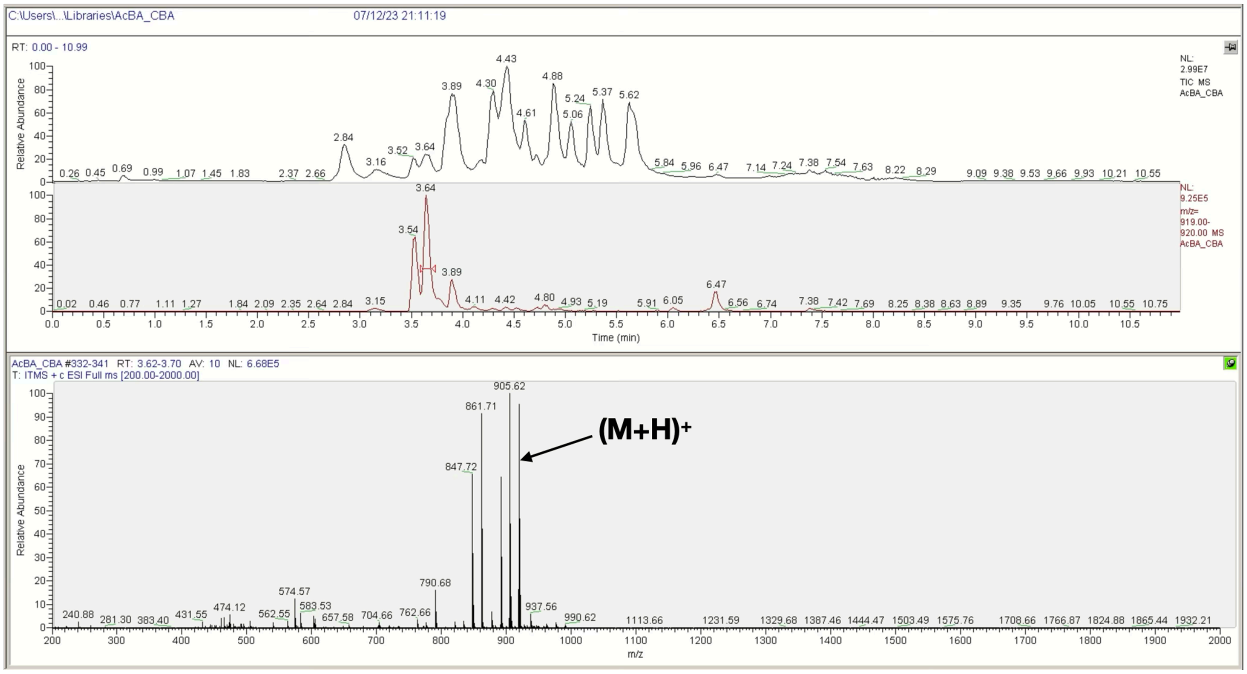

G **Ac-βA-RGEFV-Gly-βA-NH2**

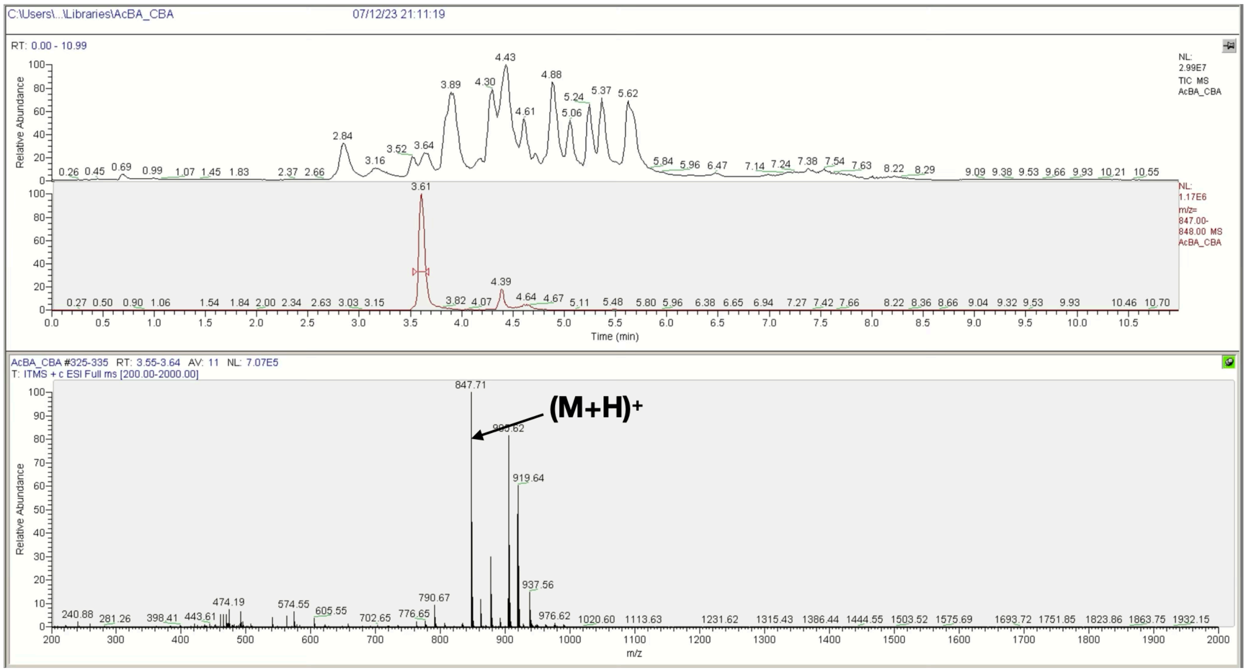

H **Ac-βA-RGEFV-His-βA-NH2**

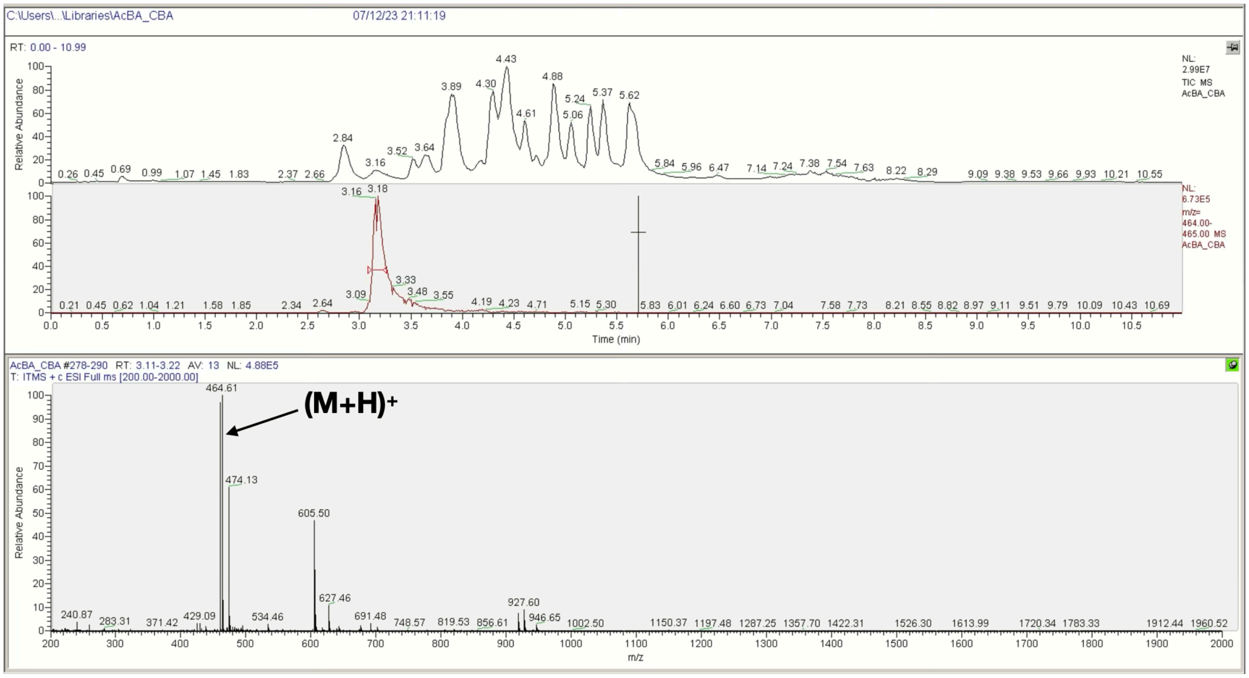

I **Ac-βA-RGEFV-Ile/Leu-βA-NH$_2$**

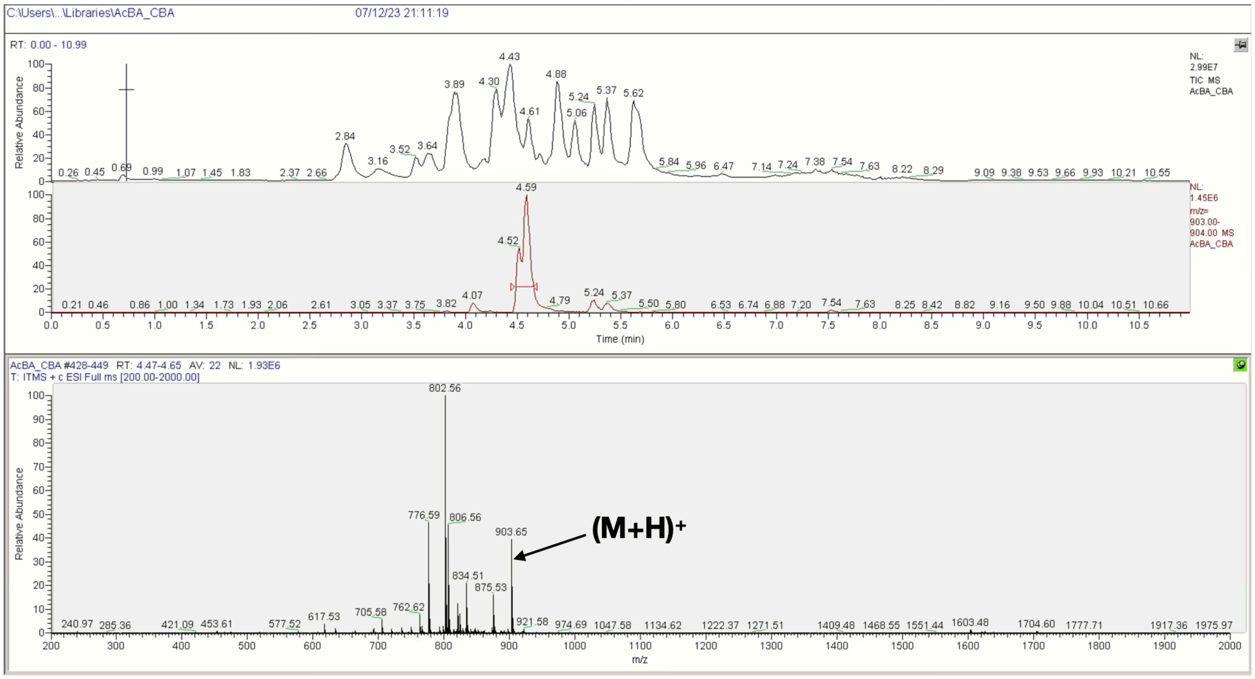

J **Ac-βA-RGEFV-Lys-βA-NH$_2$**

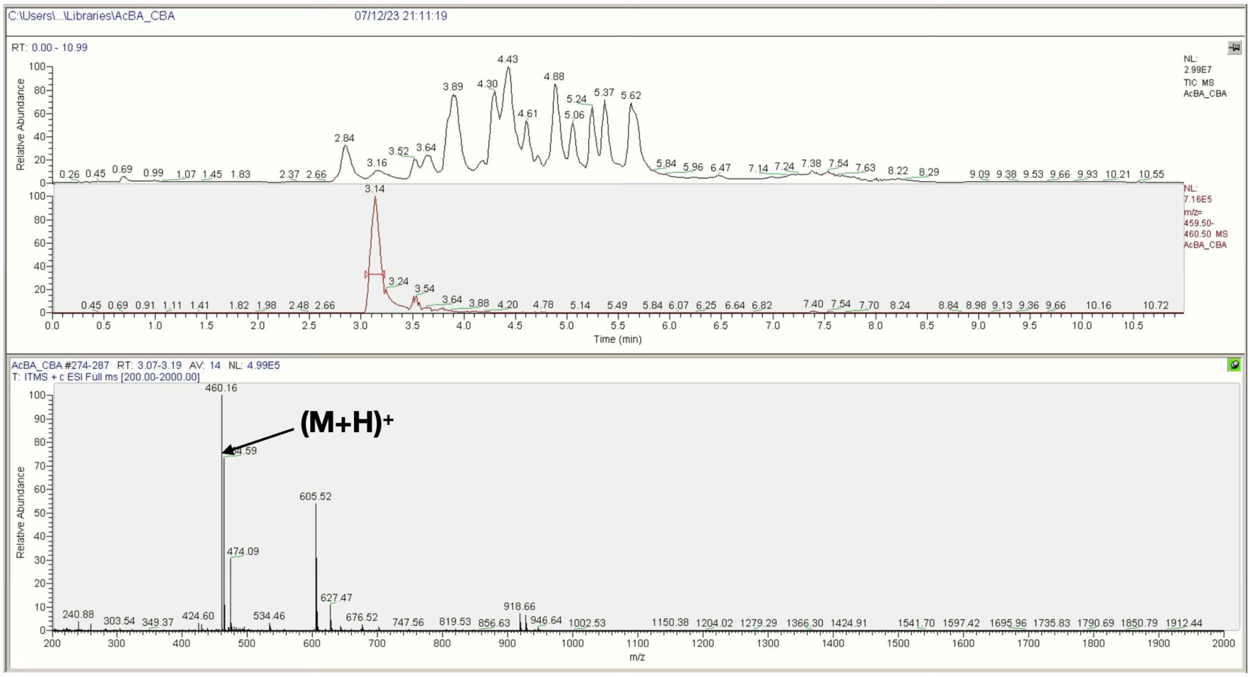

K **Ac-βA-RGEFV-Met-βA-NH$_2$**

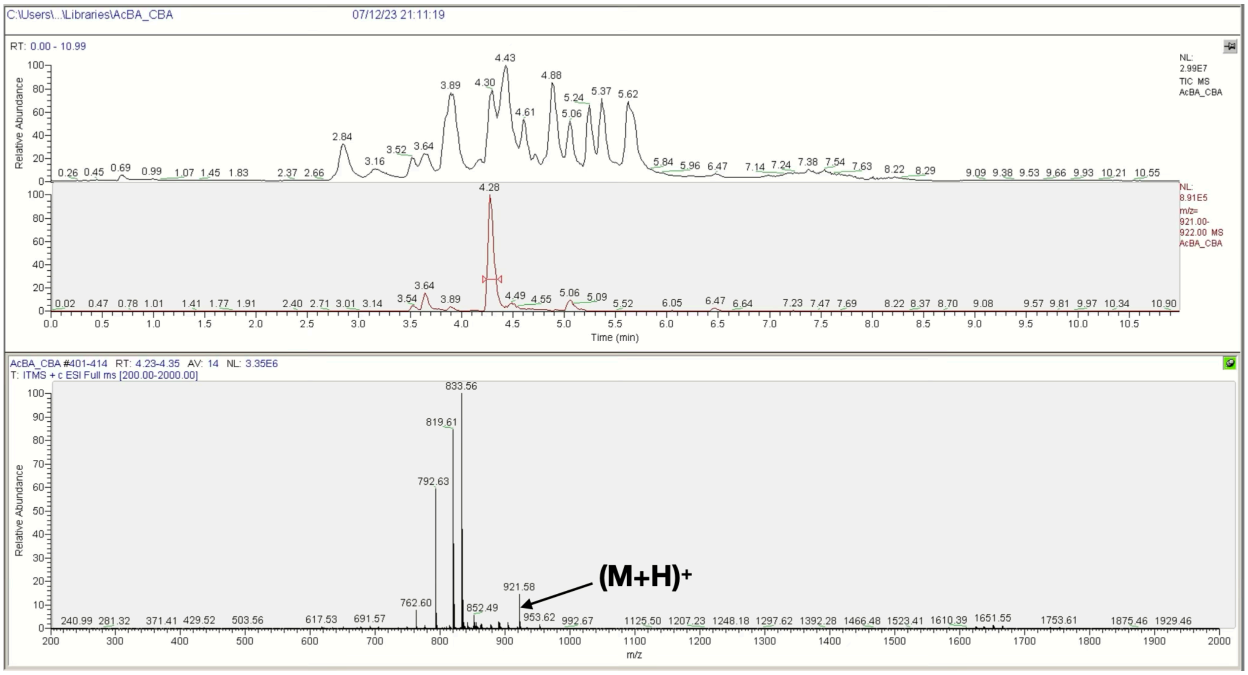

L **Ac-βA-RGEFV-Phe-βA-NH$_2$**

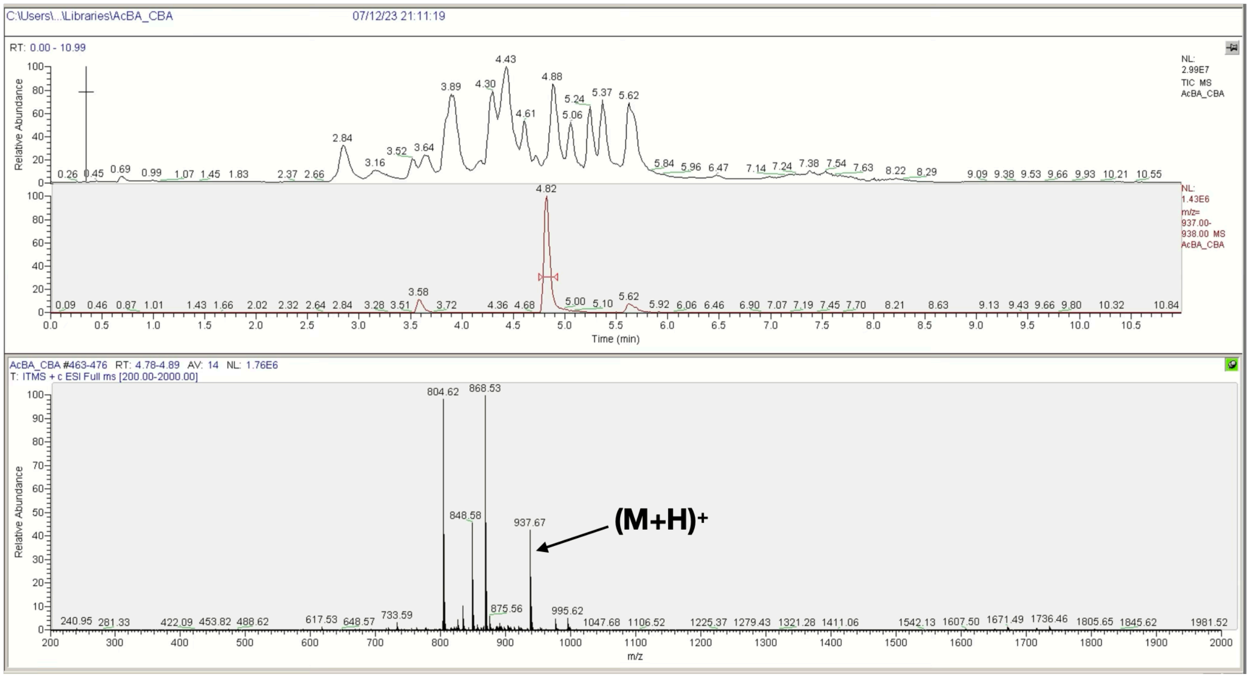

M **Ac-βA-RGEFV-Pro-βA-NH$_2$**

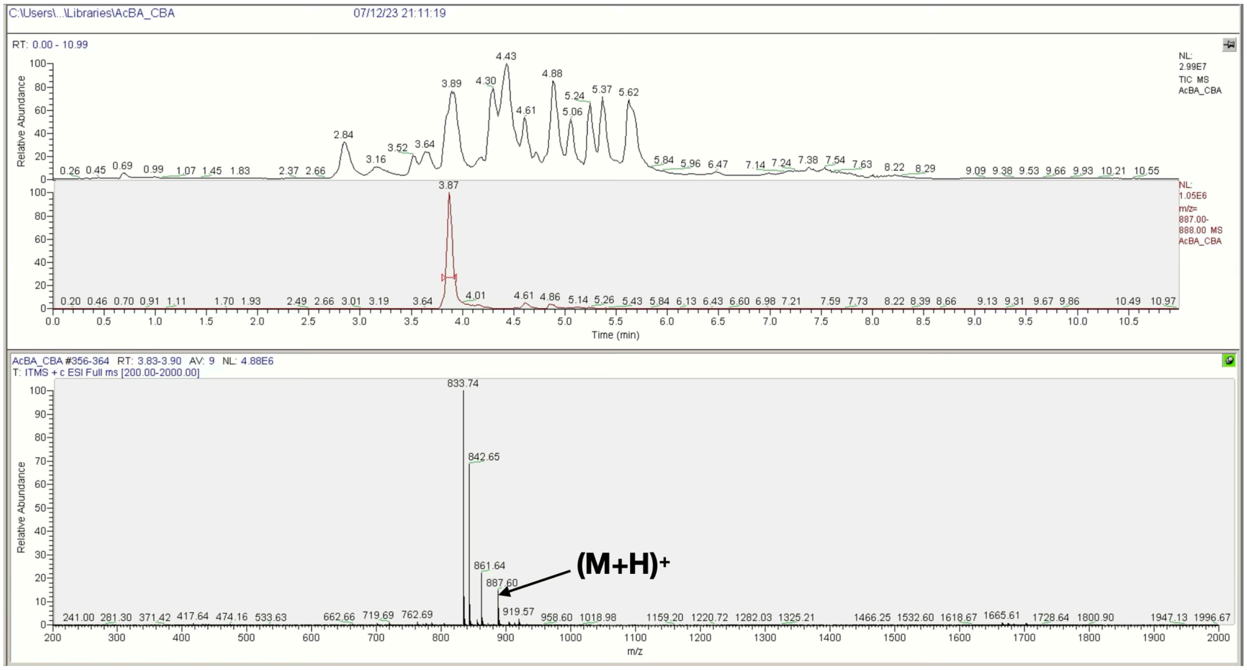

N **Ac-βA-RGEFV-Ser-βA-NH$_2$**

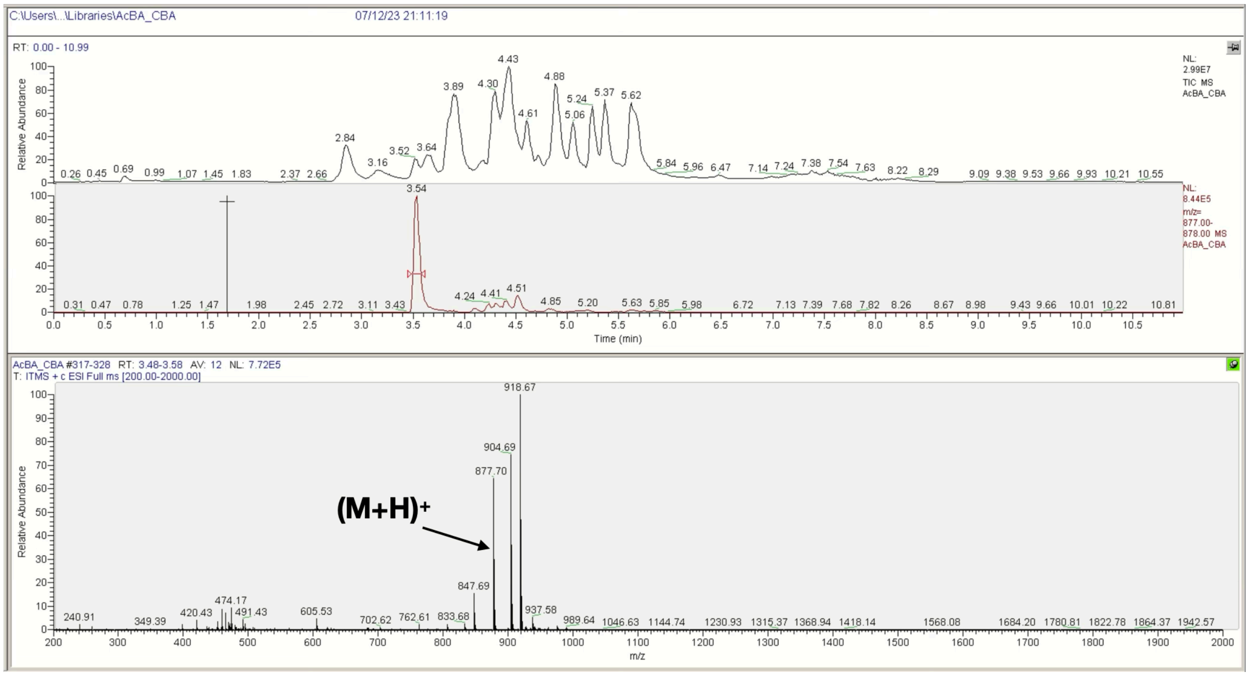

O **Ac-βA-RGEFV-Thr-βA-NH₂**

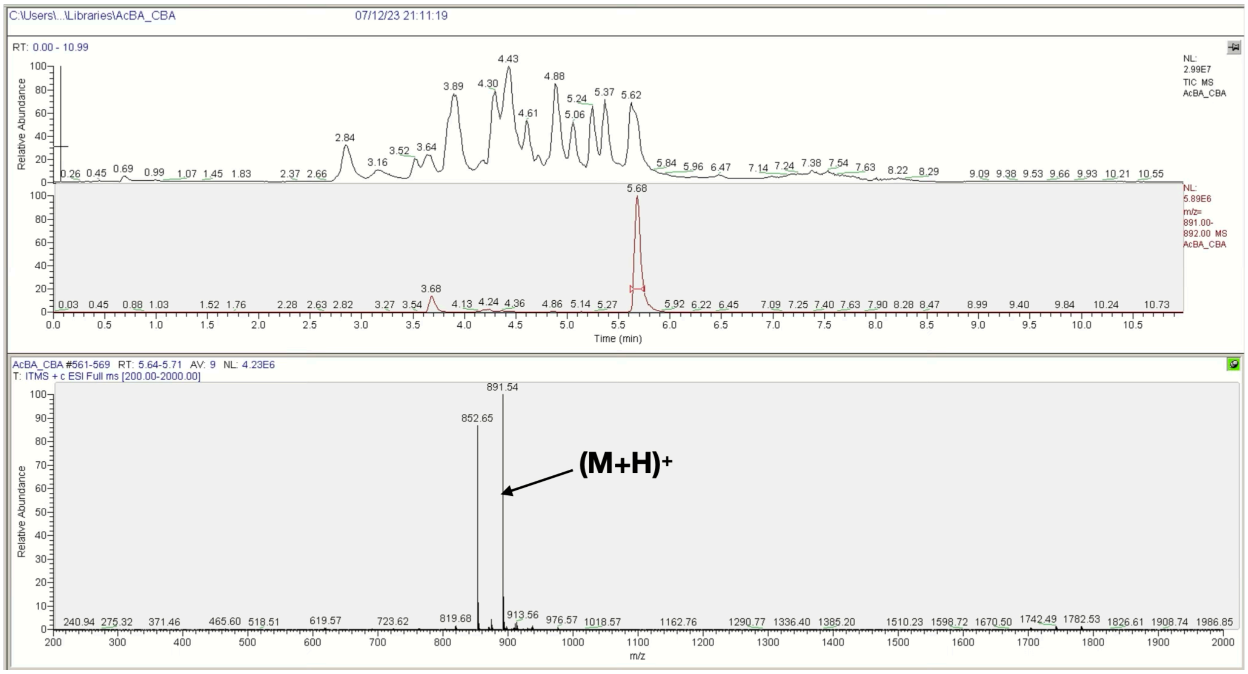

P **Ac-βA-RGEFV-Trp-βA-NH₂**

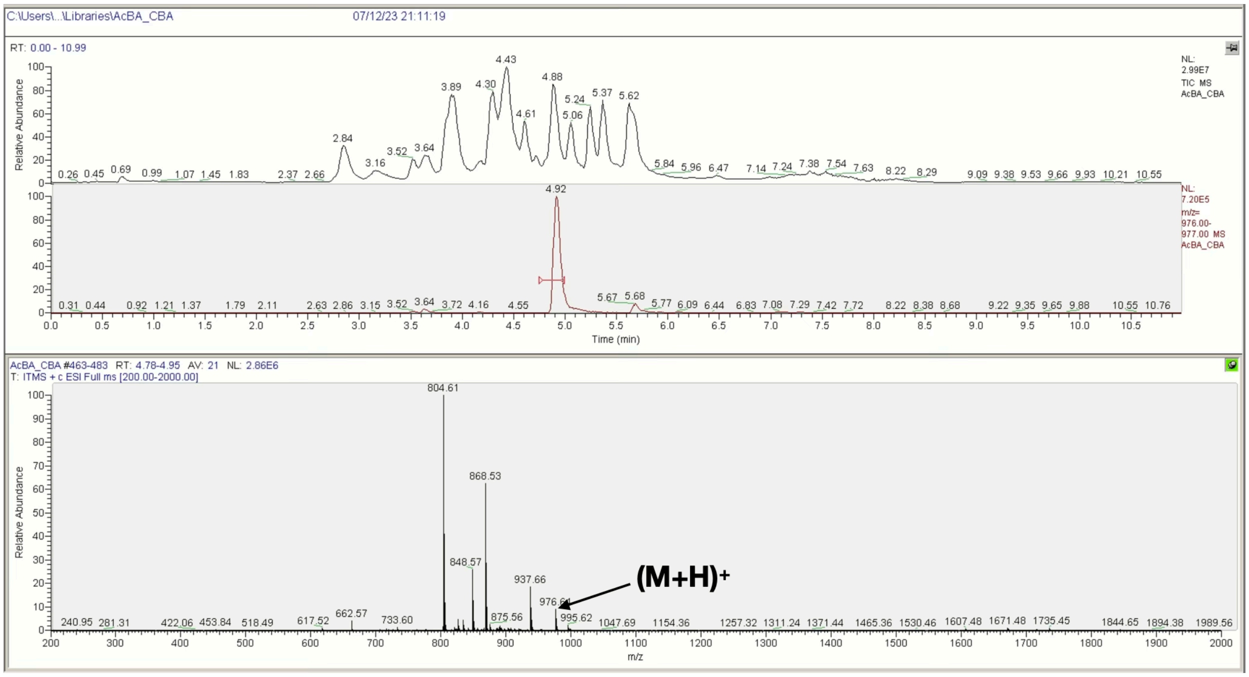

Q **Ac-βA-RGEFV-Tyr-βA-NH_2**

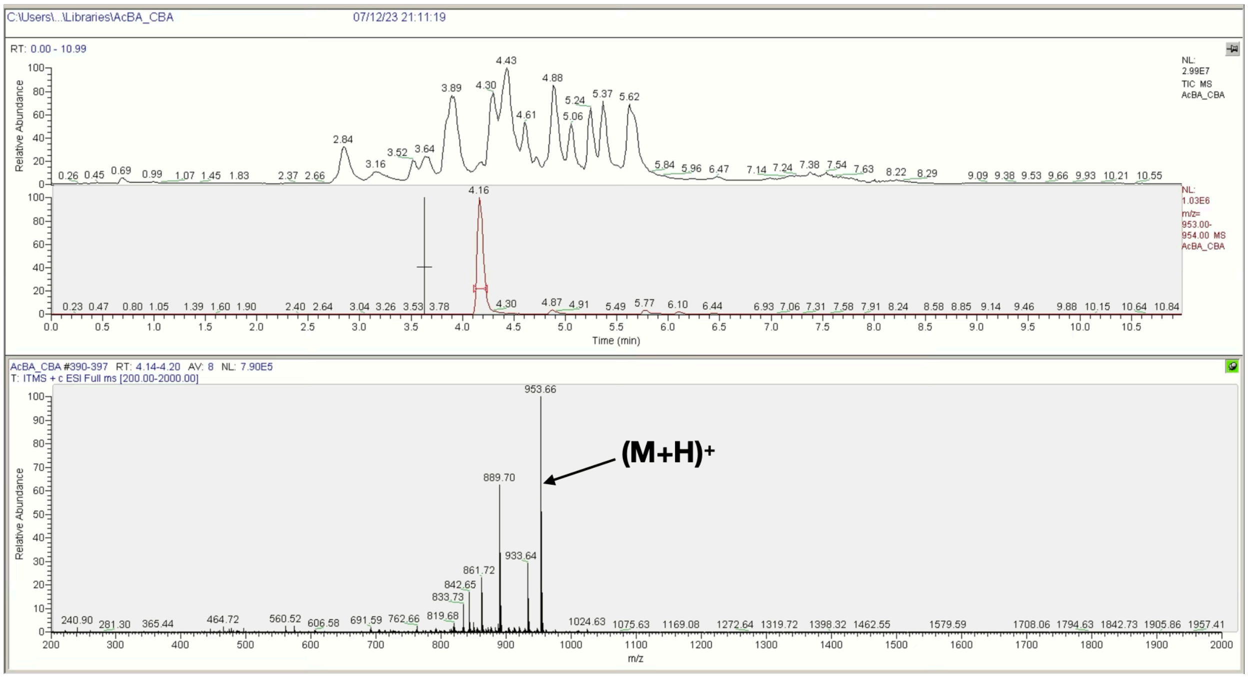

R **Ac-βA-RGEFV-Val-βA-NH_2**

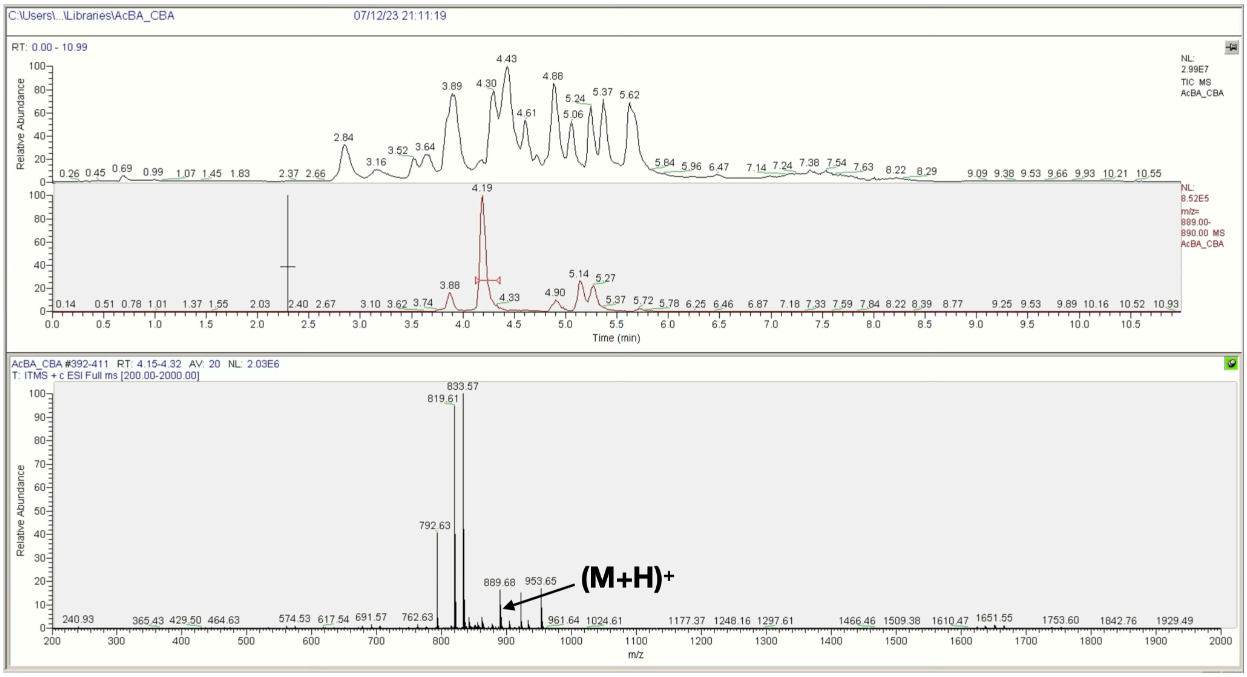

Figure S3. LCMS spectra of the Ac-βA-RGEFV-X-NH_2 libraries, where X = a) Ala, b) Arg, c) Asn, d) Asp, e) Gln, f) Glu, g) Gly, h) His, i) Ile/Leu, j) Lys, k) Met, l) Phe, m) Pro, n) Ser, o) Thr, p) Trp, q) Tyr, r) Val.

NH_2-βF-(βA)$_6$-Amide

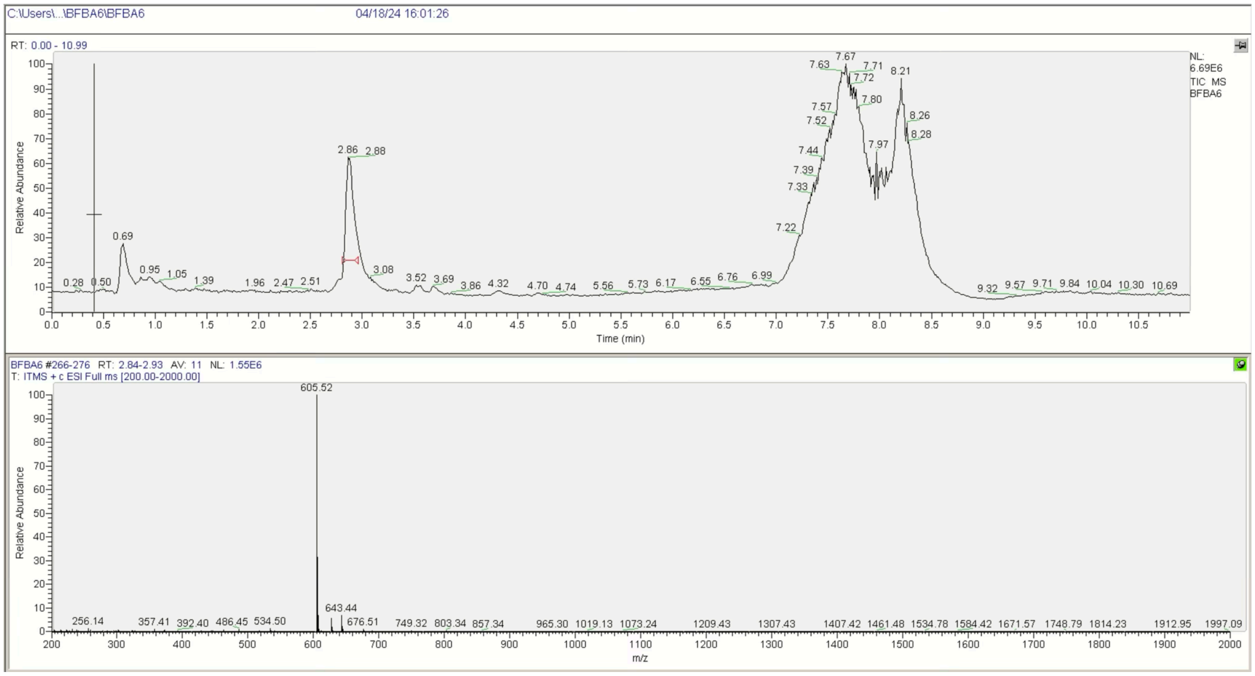

Figure S4. LCMS spectra of the internal standard NH_2-(βF)-(βA)$_6$-amide.
